# The Molecular Basis of Gender Variations in Mortality Rates Associated With the Novel Coronavirus (COVID-19) Outbreak

**DOI:** 10.3389/fmolb.2021.728409

**Published:** 2021-09-17

**Authors:** Ibrahim Y. Hachim, Mahmood Y. Hachim, Iman Mamdouh Talaat, Vanessa M. López-Ozuna, Narjes Saheb Sharif-Askari, Saba Al Heialy, Rabih Halwani, Qutayba Hamid

**Affiliations:** ^1^ Clinical Sciences Department, College of Medicine, University of Sharjah, Sharjah, United Arab Emirates; ^2^ Sharjah Institute for Medical Research, University of Sharjah, Dubai, United Arab Emirates; ^3^ College of Medicine, Mohammed Bin Rashid University of Medicine and Health Sciences, Dubai, United Arab Emirates; ^4^ Department of Pathology, Faculty of Medicine, Alexandria University, Alexandria, Egypt; ^5^ Woman’s Breast Health Centre, Ottawa Hospital Research Institute, Ottawa, ON, Canada; ^6^ Prince Abdullah Ben Khaled Celiac Disease Research Chair, Department of Pediatrics, Faculty of Medicine, King Saud University, Riyadh, Saudi Arabia; ^7^ Meakins-Christie Laboratories, McGill University, Montreal, QC, Canada

**Keywords:** gender, transcriptomics, COVID-19, ras, hydrolase activity, sex-based immunological differences

## Abstract

Since the outbreak of the novel coronavirus disease (COVID-19) at the end of 2019, the clinical presentation of the disease showed a great heterogeneity with a diverse impact among different subpopulations. Emerging evidence from different parts of the world showed that male patients usually had a longer disease course as well as worse outcome compared to female patients. A better understanding of the molecular mechanisms behind this difference might be a fundamental step for more effective and personalized response to this disease outbreak. For that reason, here we investigate the molecular basis of gender variations in mortality rates related to COVID-19 infection. To achieve this, we used publicly available lung transcriptomic data from 141 females and compare it to 286 male lung tissues. After excluding Y specific genes, our results showed a shortlist of 73 genes that are differentially expressed between the two groups. Further analysis using pathway enrichment analysis revealed downregulation of a group of genes that are involved in the regulation of hydrolase activity including (CHM, DDX3X, FGFR3, SFRP2, and NLRP2) in males lungs compared to females. This pathway is believed to be essential for immune response and antimicrobial activity in the lung tissues. In contrast, our results showed an increased upregulation of angiotensin II receptor type 1 (AGTR1), a member of the renin-angiotensin system (RAS) that plays a role in angiotensin-converting enzyme 2 (ACE2) activity modulation in male lungs compared to females. Finally, our results showed a differential expression of genes involved in the immune response including the NLRP2 and PTGDR2 in lung tissues of both genders, further supporting the notion of the sex-based immunological differences. Taken together, our results provide an initial evidence of the molecular mechanisms that might be involved in the differential outcomes observed in both genders during the COVID-19 outbreak. This maybe essential for the discovery of new targets and more precise therapeutic options to treat COVID-19 patients from different clinical and epidemiological characteristics with the aim of improving their outcome.

## Introduction

Since the outbreak of the novel coronavirus disease (COVID-19) in the end of 2019, this disease has become a public health emergency with a global impact that attracts international interest (Wenham et al., 2020). While most of the COVID-19 patients were found to suffer from only mild to moderate symptoms, the other 19% of patients suffer from a more severe disease which in some cases progress to a critical condition (Wu and McGoogan, 2020). Interestingly, the fatality rate showed great variability between various populations as well as risk groups. This was attributed to several factors including age, presence or absence of co-morbidities like obesity, diabetes, cardiovascular diseases as well as chronic respiratory diseases (Caramelo et al., 2020). For that reason, better understanding and early identification of risk factors that might predispose to more aggressive clinical course may be essential for the adoption of more effective management strategies including early intensive care and more personalized therapeutic options.

Interestingly, while several reports from different parts of the world revealed an equal distribution of cases between men and women, the mortality rate showed a significant difference between both genders with men forming around two-thirds of the deceased patients compared to only one third of women (Huang et al., 2020; Epidemiology Working Group for NCIP, 2020; Zhou et al., 2020).

This difference in mortality rates can also be attributed to gender-related factors including hormonal variations. This was supported by *in vivo* studies which showed that ovariectomized females in addition to male animals had higher levels of ACE2 activity compared to non-ovariectomized females. Indeed this indicates the possible role of sex hormones in regulation of ACE2 activity Walter and McGregor (2020), which was found to be essential for the COVID-19 virus binding and entry to the host cells in both upper and lower respiratory tracts (Markus, 2020).

Social and behavioural differences such as smoking and alcohol consumption, which are closely associated with comorbidities including cardiovascular and lung diseases The Lancet (2020), were also proposed as essential factors that might contribute in the variable mortality rates between both genders. Sex-based immunological differences were also suggested to play a role in this variation (Chen et al., 2020). For example, while the IgG antibody levels in both genders were found to be similar in the mild cases of COVID-19, female patients with severe cases showed a significantly higher levels of SARS-CoV-2 IgG antibodies compared to male patients (Zeng, 2020).Other mechanisms that were proposed to play a role in the higher vulnerability rate of males in COVID-19 pandemic is the gender-defined genetic polymorphisms and molecular variations (Zeng, 2020).

While these observations showed a possible role of gender-related genetic and molecular variations in determining the clinical behaviour of the disease including the higher mortality rate in male patients compared to females, the full mechanisms underlying such differences still need to be more clarified.

Indeed, a better understanding of the molecular mechanisms that differentially affect males and females leading to variable infection vulnerability as well as mortality is essential for the discovery of novel pathways and targets. This might help in the implementation of new therapeutic options aiding in a more effective, personalized, and comprehensive approach to treat the COVID-19 outbreak.

## Methods

### Differential Expressed Genes (DEGs) Between Males’ and Females’ Lung Tissues

**FIGURE 1:**
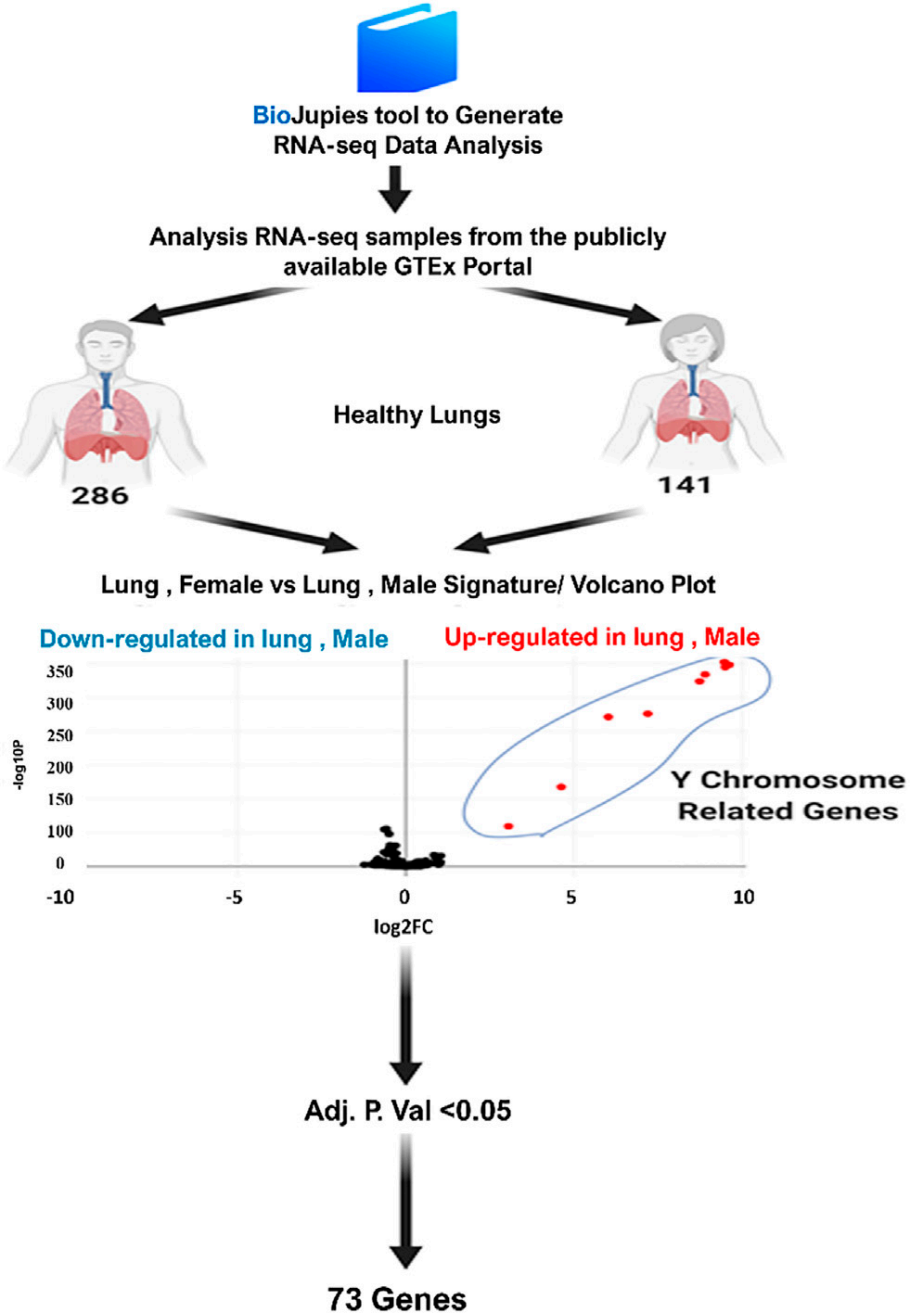
Schematic representation to bioinformatics analysis of differentially expressed genes in males compared to females’ lung tissues using BioJupies tools (Caramelo et al., 2020).

To have a better understanding of the molecular basis of the variable gender-related response to COVID-19 infection and due to the fact that the lung tissue damage is an essential event in the disease pathogenesis, we investigated the differentially expressed genes (DEGs) from lung tissues obtained from males and females using publicly available database (https://www.gtexportal.org/home/) BioJupies tool (Torre et al., 2018). A schematic representation of the bioinformatics analysis is shown in Figure 1.

### Enriched Ontology Clustering of the Identified DEGs

Enriched Ontology Clustering for the identified genes was performed to explore if the identified genes are sharing common pathways using the Metascape (a web-based tool used for comprehensive gene list annotation and analysis resource) Zhou et al. (2019), as shown in Figure 1.

### Deciphering Organ and Sex-specific Gene Expression Levels Variation

The Genotype-Tissue Expression (GTEx) project (www.gtexportal.org) was used to evaluate the variation in the gene expression levels of the selected genes according to sex and organs including the lung and kidney (Consortium, 2017). This platform includes the human transcriptomic data across different individuals and allow researchers to investigate the reference values of the gene expression levels for a range of normal primary tissues and organs. In addition, it allows the stratification of the gene expression levels with some clinical parameters including gender.

The average expression of our candidate genes was evaluated across different cell population of human lung tissue using Lung Gene Expression Analysis Web Portal “LungGENS” (https://research.cchmc.org/pbge/lunggens/), which is a web-based tool used to investigate the expression levels of different genes in single-cell population.

### Statistical Analysis

For the shortlisted DEGs, we select 2-fold change as a threshold between different groups and adjusted *p* value < 0.05 was used as cut-offs.

For the LungGENS, we used the “Query by single gene” tool. The search engine in that database use *t* test to compare the expression levels of the selected genes among the different cell types. Further analysis was done using pie chart to show the percentage of each gene and its expression in each cell type using the *p* value < 0.05 as a cut-off.

## Results

Lung tissues from both genders showed differential expression of 73 genes in male and female lung tissues.

Initially, we compared the lung transcriptomic data of 141 females with 286 males. Our results showed 85 genes with significant variation in the expression levels between the two groups (Table 1). After excluding Y specific genes, 73 genes were selected.

**TABLE 1:** Top differential genes between males’ and females’ lung tissues.

DDX43	KCNIP3	GEMIN8	SLC2A1	ITGAD	EFHC2	SYTL5
SFRP2	SLC4A3	CA5B	LANCL3	STS	KDM6A	
OOEP	AGTR1	RHOH	ZRSR2	RPS4X	PNPLA4	
GRM8	KRBOX1	NAP1L2	KDM5C	RNF183	ERCC6L	
NOX5	MRC2	PLIN4	FAM3B	UGT8	RIBC1	
SPESP1	MAN2C1	SMC1A	CEACAM6	SRRM4	LYPD6B	
AJAP1	CHM	EIF2S3	DDX3X	ARSD	AQP5	
FAM228A	TRAPPC2	SYAP1	GYG2	TNFRSF13B	NLRP2	
PTGDR2	UBA1	FGFR3	ADD2	CP	BEND2	
GPAT2	ZDHHC2	TXLNG	KEL	ZFX	MAP7D2	
MMEL1	EIF5	PRKX	EIF1AX	PCDHA1	SAA4	
PRPH2	OFD1	FBXL16	PLEKHG4B	HS6ST2	SAA2	

Significant pathways in which the identified DEGs are differentially expressed between males’ lung tissues compared to females’

**FIGURE 2:**
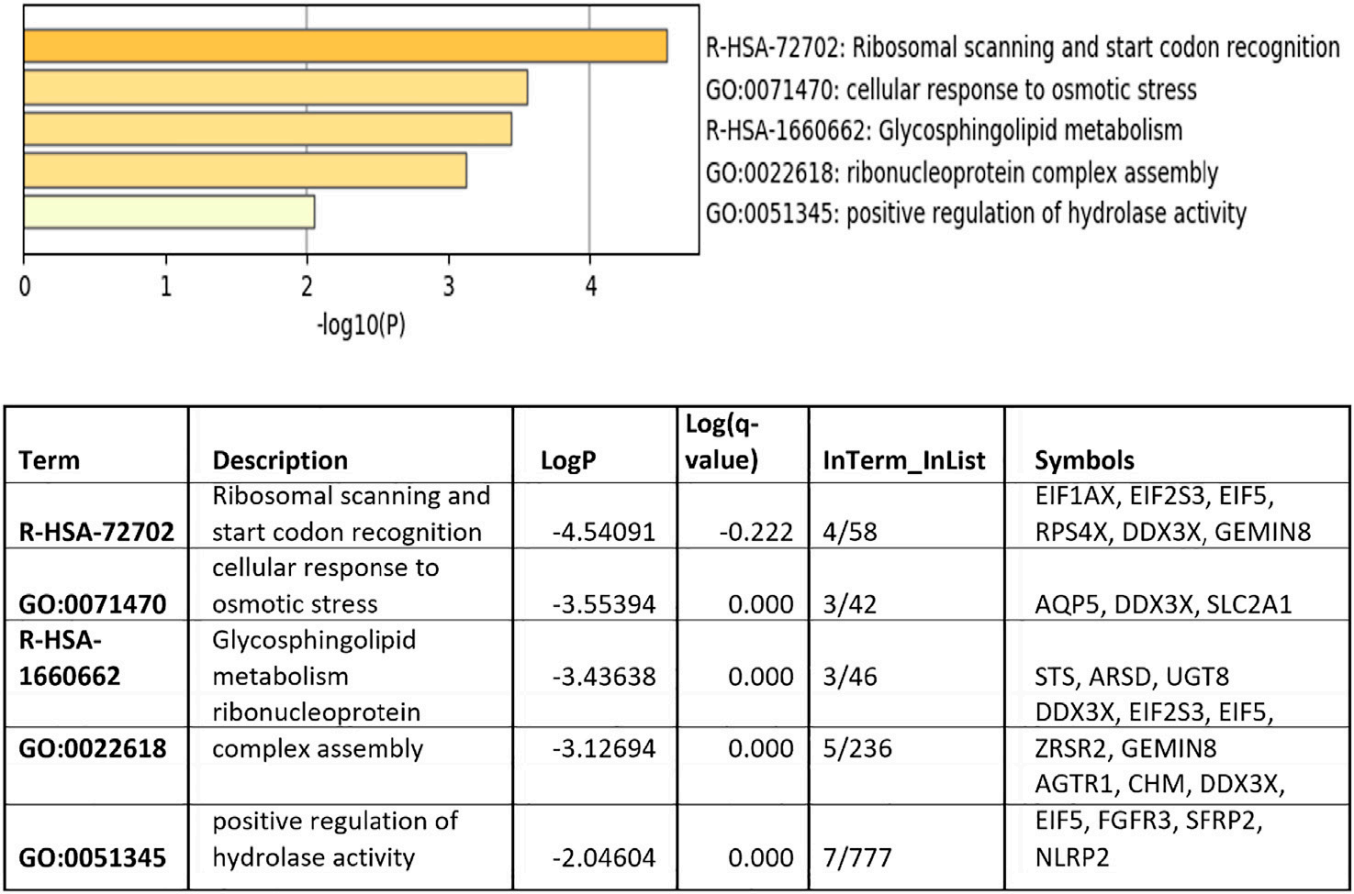
Top pathway enrichment for the differentially expressed genes between males’ and females’ lung tissues. Genes related to the hydrolase activity are enriched in females’ lung tissue compared to males’.

Further analysis of the DEGs that differentiated between males’ and females’ lung tissues revealed that our top differential genes are enriched with several pathways. These include a pathway related to positive regulation of hydrolase activity (AGTR1, CHM, DDX3X, FGFR3, SFRP2, and NLRP2), which is believed to be important in the lung physiology as well as inflammation. Pulmonary surfactant contains homeostatic and antimicrobial hydrolases that are found to play a significant role in the terminal bronchioles and alveoli microbes control (Arcos et al., 1950) (Figure 2). Moreover, some of our top differentially expressed genes belong to the glycosphingolipid metabolism pathway. Reports highlighted that this pathway is essential in the virus–host interactions through regulating the ability of viruses to bind to gangliosides and determining the internalization pathway into cells.

**FIGURE 3:**
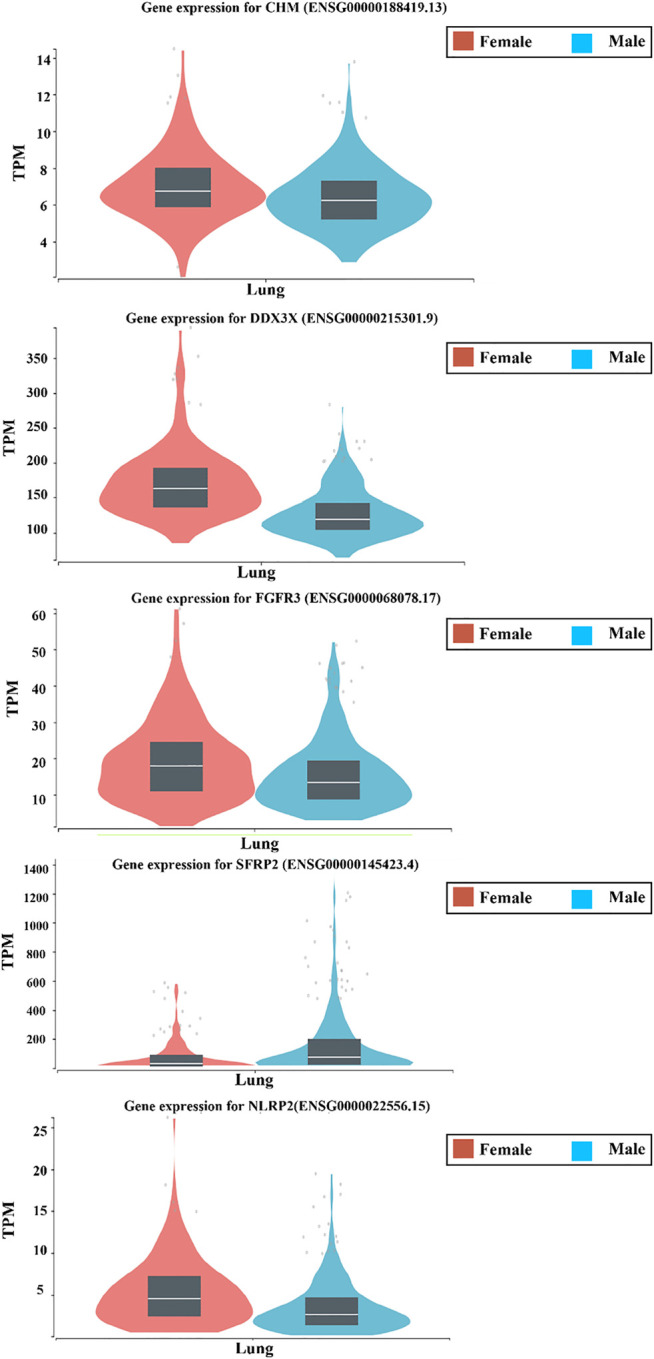
The expression level of different genes related to the lung hydrolase activity between males’ and females’ lung tissues using The Genotype-Tissue Expression (GTEx) project (www.gtexportal.org) portal.

Previous reports showed a possible role of several hydrolases in human lung tissues and alveolar lining fluid, in the modulation of microorganism envelope suggesting their possible role in infection control (Arcos et al., 1950). For that reason, we try to further validate the expression levels of our top differentiated genes involved in the lung hydrolase activity using The Genotype-Tissue Expression (GTEx) project (www.gtexportal.org) portal, which is another independent publicly available database. Interestingly, our results showed that CHM, DDX3X, FGFR3, and NLRP2 are more expressed in lung tissues obtained from female’s lungs compared to males. This further highlights a possible role of these genes in controlling the COVID-19 infection through regulating the lung hydrolases activity (Figure 3).

Angiotensin II type 1 receptor (AGTR1) is among the differentially expressed genes in both genders and its expression is significantly higher in the lung tissue compared to the kidney tissue.

**FIGURE 4:**
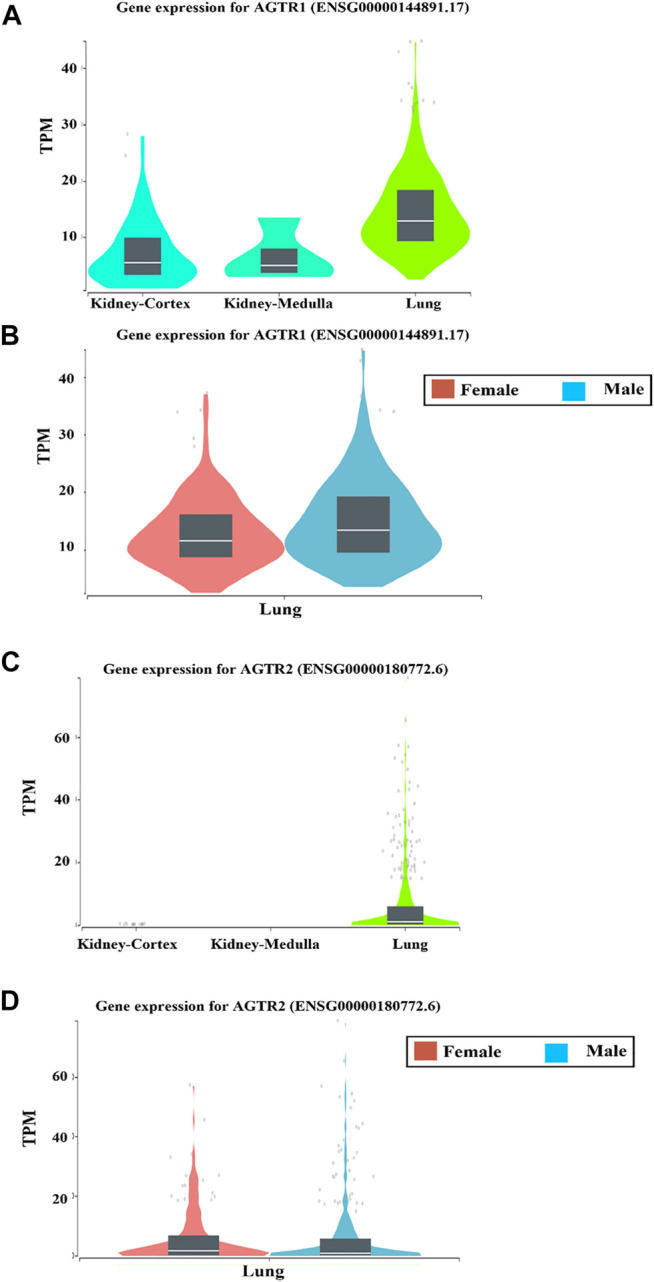
The mRNA expression levels of AGTR1 and AGTR2 in lung and kidney tissues using The Genotype-Tissue Expression (GTEx) project (www.gtexportal.org) portal.The prostaglandin D2 receptor 2 (PTGDR2), involved in type 2 innate immune response and well known for its role in airway inflammation is another gender differential gene in the lung tissue.

Our short-listed genes also revealed the presence of AGTR1 among the gender-related differentially expressed genes. Interestingly, AGTR1 is one of the 2 G protein-coupled receptors essential for the physiological effects of angiotensin II (ANG II). Since many reports highlighted that the role of human receptor ACE2 belongs to the renin-angiotensin system (RAS) and facilitates the binding of the SARS-CoV-2 to host cells, we further analysed the mRNA expression levels of AGTR1 and AGTR2 in lung tissue compared to kidney tissue as an example of other tissues, using The Genotype-Tissue Expression (GTEx) project (www.gtexportal.org) portal. Our results showed that both receptors were shown to be upregulated in the lung tissue compared to the kidney tissue (Figures 4A, C). Next, we analysed their expression in the lung tissues from both genders. Interestingly, the results revealed that while AGTR2 showed no significant difference in its expression in both genders, AGTR1 expression levels were higher in tissues obtained from male individuals compared to females (Figures 4B,D). This further supports our initial finding that AGTR1 is among the top differential genes in both genders.

**FIGURE 5:**
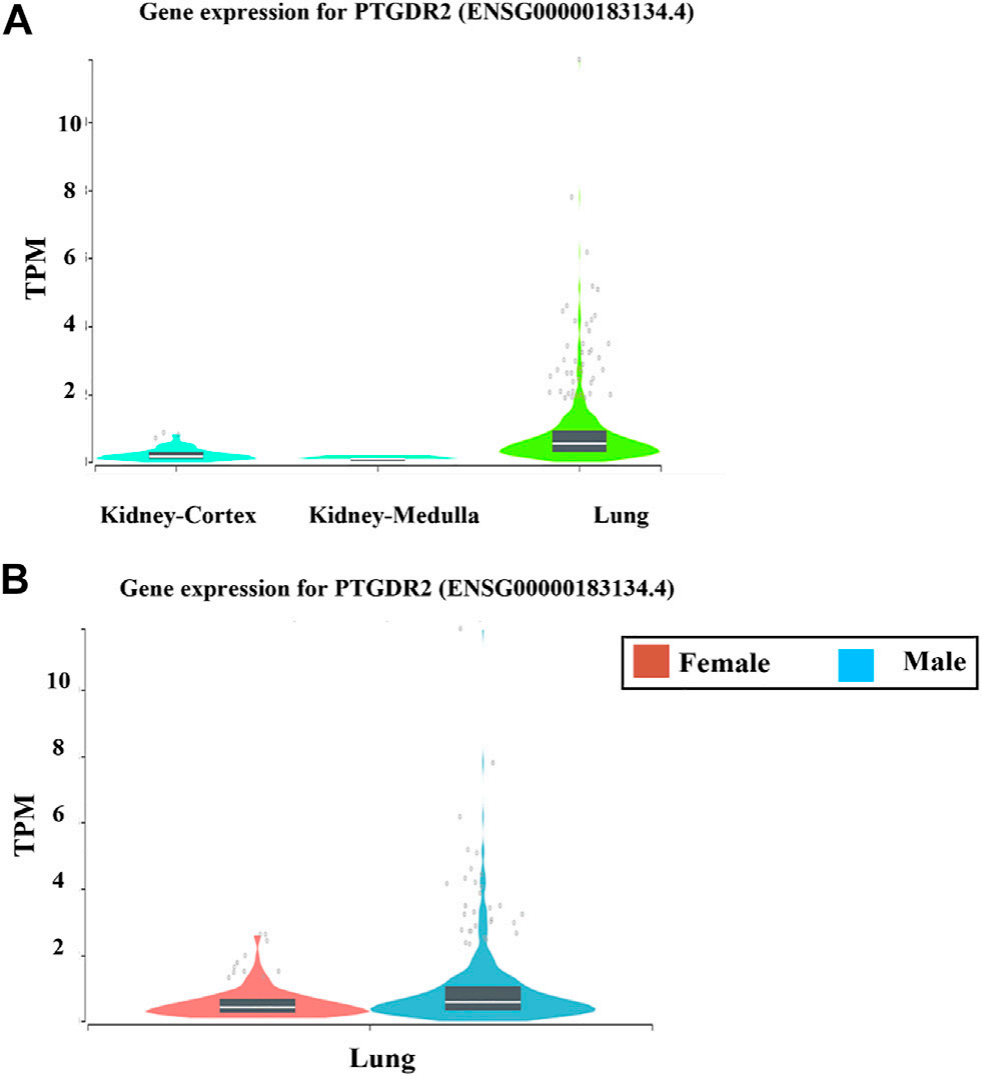
The mRNA expression levels of PTGDR2 in lung and kidney tissues using The Genotype-Tissue Expression (GTEx) project (www.gtexportal.org) portal Cellular localization of the differentially identified gene candidates.

Another interesting gene that we also found in our short list is the prostaglandin D2 receptor 2 (PTGDR2). This gene is essential for cells involved in type 2 immune responses through its interaction with prostaglandin D2 (PGD2). Also, it is known to play an essential role in the pathogenesis of asthma, through its role in induction of the pro-inflammatory cytokines and cationic proteases. Our analysis also revealed that PTGDR2 expression is higher in the lung tissue compared to the kidney tissue (Figure 5A). Moreover, its expression was found to be upregulated in male lungs compared to females’ (Figure 5B). This further confirms our initial findings.

**FIGURE 6:**
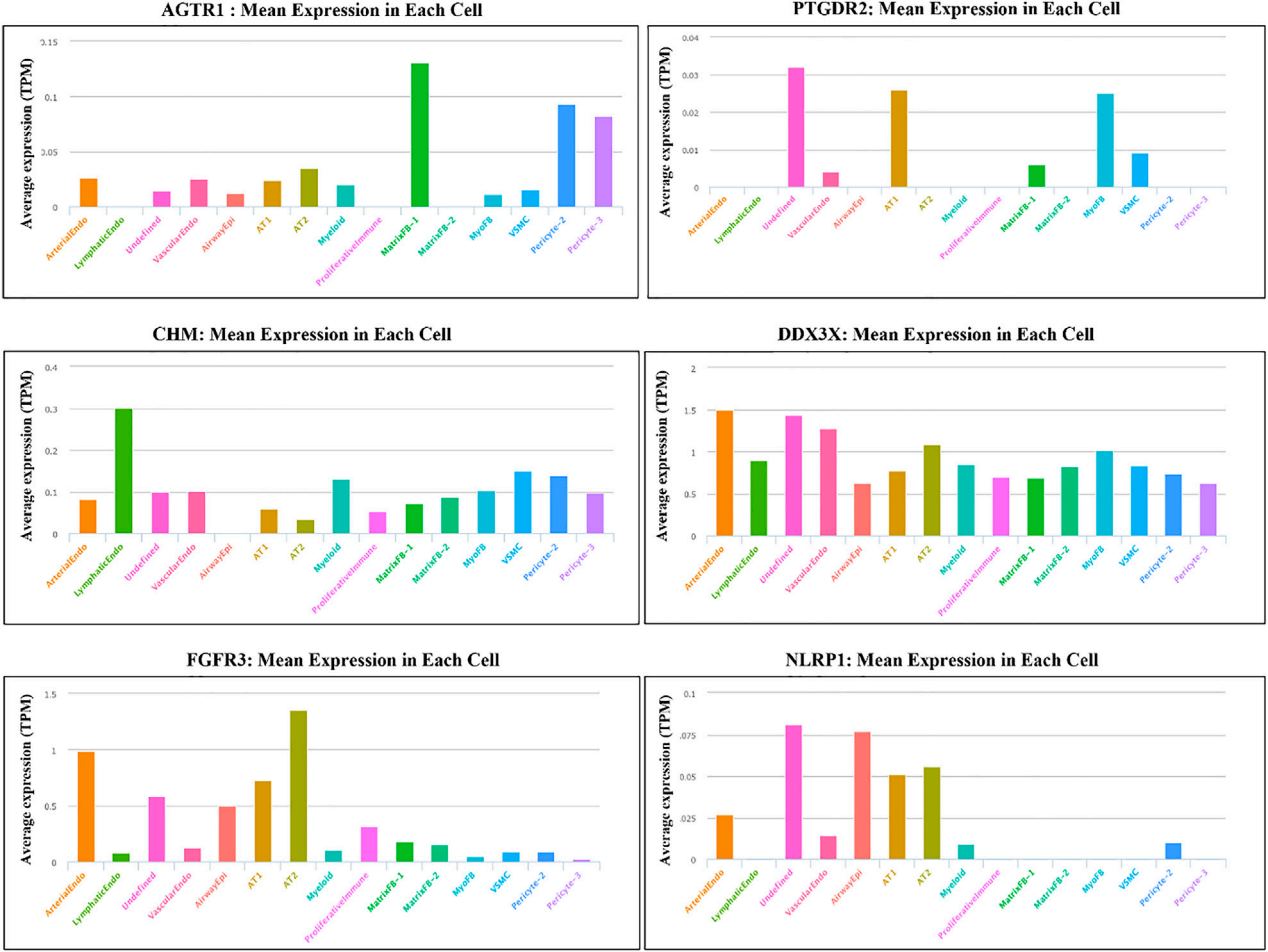
The mRNA expression levels of different candidate genes in different lung cell populations using Single-cell RNA analysis of LungGENS web-based tool.

To have a better idea of the cellular localization of our short listed genes in the lung tissue, we investigated the gene expression levels of those genes using single-cell profiling of human lung tissues (the LGEA portal: https://research.cchmc.org/pbge/lunggens/mainportal.html) (Figure 6). Our results showed a variable expression of the candidate genes in different cell populations. Interestingly, all genes were expressed in the alveolar type I (AT1) and alveolar type II (AT2) cells in the respiratory system. FGFR3, PTGDR2 and NLRP2 were predominately expressed in the epithelial cells of the respiratory system including the airway epithelial cells, alveolar type I (AT1) and alveolar type II (AT2). In contrast, AGTR1 showed a predominant expression in the matrix fibroblasts and pericytes compared to other cells.

## Discussion

Despite the fact that COVID-19 is characterized by a milder clinical course of disease for the majority of patients compared to other coronavirus infections like SARS‐CoV and MERS‐CoV Lukassen et al. (2020), patients belonging to specific ethnic and demographic subgroups and those presented with pre-existing comorbidities showed higher rates of serious adverse outcomes including high mortality rates compared to the general population (Chen et al., 2020; Guan, 2020; Guan et al., 2020; Huang et al., 2020; Pareek et al., 2020).

For that reason, the identification of different risk factors that predisposes for adverse outcome and the discovery of the molecular pathways that promote such severe course might be an essential step to provide more effective management plans Pareek et al. (2020), in addition to the discovery of new targets and tools that may lead to the adoption of more precise targeted therapies.

One of the striking findings related to COVID-19 outbreak is the significant difference in the mortality rates between male and female patients despite the equal numbers of infection for both genders (Epidemiology Working Group for NCIP, 2020). According to the available reports, elderly male patients with comorbidities are more likely to die from COVID-19 compared to females in a ratio reaching to 3:1 (Di Stadio et al., 2020; Huang et al., 2020; Epidemiology Working Group for NCIP, 2020; Wenham et al., 2020; Zhou et al., 2020).

The difference observed between both genders was suggested to be associated with some social and behavioural habits including smoking and alcohol consumption The Lancet (2020) as well as sex-based immunological differences between both genders (Chen et al., 2020; Zeng, 2020). Despite all these preliminary findings, there is no in-depth analysis to understand the genetic and molecular basis of this difference.

The here presented data sheds the light on some possible molecular pathways that might be responsible for this gender difference. Our results showed a downregulation of genes that are involved in regulating hydrolase activity including (CHM, DDX3X, FGFR3, and NLRP2) in the lung tissues obtained from males compared to that obtained from females. Interestingly, recent reports linked the hydrolase activity with lung immune response and inflammation as well as to antimicrobial effect (Arcos et al., 2017; Arcos et al., 1950). Moreover, alveolar lining fluid (ALF) hydrolases was found to be involved in the regulation of macrophages function as well as the host immune response, essential for infection control (Arcos et al., 1950; Arcos et al., 2017; Scordo et al., 2017). For that reason, the downregulation of genes involved in this pathway in male’s lung tissues might explain the higher vulnerability of male patients to more severe clinical course.

Another important finding in this study, is the upregulation of the AGTR1 gene (also known as AT1 receptor), in the lung tissue obtained from males compared to that obtained from females. This gene is considered as a fundamental component of the renin-angiotensin system Mottl et al. (2008) and discovered to have strong interaction with the ACE2. Indeed, the fact that ACE2 is considered as an essential component mediating COVID-19 virus entry to the human respiratory cells Mottl et al. (2008) may highlight a possible specific role of AGTR1 in the ACE2 mediated binding of the COVID-19 to the host cells in male patients. This suggestion is supported by recent findings that blockage of AGTR1 with losartan in experimental models helped in reducing the pulmonary edema as well as the severe acute lung injury associated with SARS-Coronavirus infection (Kuba et al., 2005; Gurwitz, 2020). For that reason, researchers suggested the use of AGTR1 (AT1R) antagonists like losartan and olmesartan and other angiotensin II receptor blockers (ARBs) as a possible tentative therapeutic option for COVID-19 infection that may protect COVID-19 patients from the severe symptoms and reduce their mortality rate (Gurwitz, 2020; Sun et al., 2020).

Finally, our findings showed a differential expression of some genes that play a role in the immune response. This includes the NLRP2, which is involved in the suppression of the NF-κB signaling pathway leading to the modulation of the inflammatory response (Fontalba et al., 2007). In addition, it is considered as an important component of the inflammasome, which is a multiprotein intracellular complex, essential for the detection of the pathogenic microorganisms and activation of the pro-inflammatory cytokines including interleukin-1β (IL-1β) and IL-18 (de Rivero Vaccari et al., 2014; Rossi et al., 2019). Similarly, PTGDR2, which we found to be upregulated in male lungs compared to females, is believed to be essential for the pro-inflammatory cytokines induction as well as asthma pathogenesis (Huang et al., 2016). Moreover, recent reports highlighted prostaglandin D2 as a mediator of lymphopenia and adverse clinical course, suggesting it as a possible therapeutic target in COVID-19 patients (Gupta and Chander Chiang, 2020).

Indeed, this study is an essential proof of concept, however, further studies are needed to confirm the significance of those genes in determining the distinct clinical course between both genders in response to COVID-19 infection.

All together, our results shed the light on some of the molecular mechanisms and pathways that might predispose to the poor outcome and high mortality rates observed in male patients during the COVID-19 outbreak. Better understanding of such mechanisms might be essential in the discovery of new therapeutic approaches based on targeting those specific pathways, which in turn might improve the outcome of patients with different ethnic and epidemiological characteristics.

## Data Availability

The original contributions presented in the study are included in the article/supplementary material, further inquiries can be directed to the corresponding author.
